# The Treatment of Spinal Diseases

**Published:** 1883-05

**Authors:** Roberts Bartholow

**Affiliations:** Professor of Materia Medica and General Therapeutics in the Jefferson Medical College, Philadelphia


THE TREATMENT OF SPINAL DISEASES. A Clinical Lecture delivered at the Jefferson Medical College, Sessions of 1882–83. By ROBERTS BARTHOLOW, M.D., LL.D., Professor of Materia Medica and General Therapeutics in the Jefferson Medical College, Philadelphia.

GENTLEMEN: No thoughtful student can have failed to observe the greater attention paid to the pathology and diagnosis of spinal diseases than to their therapy. Indeed, the treatment is usually dismissed in a few words, as an unsatisfactory subject. Notwithstanding I have, myself, as a teacher of therapeutics, given rather more than ordinary attention during my lectures here to this department of the subject, I must still admit that my efforts in this direction have not been as full as the importance of the subject demands. I have now to make an effort to supply this omission—to place before you the more recent results attained in the treatment of spinal affections. By thus taking a comprehensive view of the whole field, we may the better comprehend the relation of the several parts and arrive at conclusions more accurately than by the consideration of individual maladies only.

From the therapeutical point of view, these spinal diseases may be comprehended in three groups: Acute inflammatory, chronic inflammatory, and nutritional diseases. It is of no special importance to fix the precise seat of the inflammation, for the principles underlying the therapeutical management are the same. To a just estimate of the curative effect of remedies in these inflammatory affections, a true conception of the spinal circulation is necessary. Look at this diagram. Observe the voluminous and tortuous veins of the spinal canal. To the nutritious arteries of the cord they bear the numerical proportion of four to one. In other words, if the capacity of the arterial supply be put at *one*, the venous capacity must be stated as *four*. It follows that the blood current sent into the spinal canal through the arteries must be slowed there to fill out the veins. This, you will explain, is an arrangement to facilitate the functional work of the spinal cord, under the various conditions interfering with the normal and equable blood-supply. Without stopping to consider the necessity, we are now concerned with the fact, simply. The blood-supply having this peculiar arrangement, do these anatomical conditions affect the question of remedies? I unhesitatingly answer this question in the affirmative.

One of the remedies most relied on to affect the intra-spinal circulation is ergot. I hold that its administration in acute spinal inflammation is improper, because of the peculiarity of its action. It induces an anæmia of the arterial distribution—an ischæmia, properly speaking—but the blood, thus driven from the arterial side, accumulates on the venous side; hence, it follows that while the arterial supply may be reduced, the veins of the spinal canal are distended unduly. The compression thus exerted on the cord has, as I conceive, a most hurtful influence on its nutrition, and hastens the progress of the changes inaugurated by the inflammation.

If ergot is inadmissible in acute inflammation of the intra-spinal organs, to what remedies shall we then resort? You may not be at all prepared for the statement I have to make, but, speaking from the standpoint of my personal observation, I have to say that aconite, digitalis, veratrum viride, opium, and bromide of potassium are the most useful remedies in the cases of acute spinal inflammation. Each, however, has its special range of utility. All agree in the power to limit the blood supply to the spinal canal, but in what degree, if at all, does each affect the intra-spinal venous circulation? Far more than ergot. If I may again refer to my own experience, I can recommend the use of digitalis first, and, if this disagree with the stomach, aconite. You will find it most useful to begin with the infusion of digitalis, administering from a tea to a tablespoonful every four hours, until the conditions for which it is used cease, or the stomach fails to retain it. If irritability of the stomach is a bar to its internal use, it may be effectively employed topically, the leaves being steeped in hot water, placed in a porous bag, and applied to the spine or abdomen. This remedy, you will find, will do more to restore the normal balance of the intra-spinal circulation than any other now at our command.

Next to it in point of utility is the tincture of aconite root. This must be given until the eharacteristic tingling is produced, or the pulse-rate is lowered. Acting both on the skin and kidneys, it favors the excretion of the products of inflammatory waste. Veratrum viride is not as useful as aconite—its action little extending beyond the hydrostatic effects. Opium, especially morphia, hypodermatically, becomes indispensable when pain is a pronounced feature in all cases, and experience has shown its utility in meningeal inflammation. The bromides, especially bromide of potassium, are indicated when reflex, convulsive phenomena are present; such as muscular cramp, twitchings, etc., indicating irritation of the motor tract.

Are there any data by means of which we may fix the time for the administration of the arterial sedatives? As these remedies only affect the vessels primarily, and secondarily the structure of tissue, when shall they be discontinued? In respect to this point, there is always no little indecision; but a correct conclusion may be reached by a careful consideration of those symptoms, indicating the occurrence of exudations—symptoms whether of excitation or of depression of function. So long as the symptoms of excitation—hyperæsthesia and spasm—continue, there can be no doubt that those remedies will be useful which have to do with the blood supply. When exudations occur and pressure is thereby brought to bear on the intra-spinal organs, the symptoms of depression or of arrest of function come on—anæsthesia and paresis.

Arterial depressants can do no possible good; only injury, indeed, when the local status is no longer that of hyperæmia and excited action; when the process of effusion and exudation comes on, we have to deal with depression of function. Remedies having very different powers then come into use.

Merely fluid effusion into the spinal canal is more easily disposed of than a solid exudation. Usually, however, the products of inflammation include both fluid and solid exudations. Absorption of merely fluid exudation may be affected by a judicious combination of purgatives and diaphoretics, especially of Epsom salts and pilocarpine. The disposition of solid exudations is more difficult. Considerable experience with the use of ammonia and its salts, especially of the acetate and carbonate, has given me very positive confidence in the power of this remedy. The most convenient mode of administering it is to dissolve the carbonate in the officinal *liquor ammonii acetatis*, so that five grains of the former will be given in a tablespoonful of the latter. At or about the time when the symptoms of depression, due to the pouring out of an exudation, come on, the solution of ammonia should be administered. The important point is to so alkalinize the blood, that local thrombus and solid exudations may be reabsorbed. To accomplish this result, it is necessary to keep the blood well alkalinized. Although this may fail, it is surely the most promising expedient in such cases, and experience has proved its utility.

If the physician is so fortunate as to see the case at the very moment of its inception, the best results are to be expected from the administration of a maximum dose of quinine and morphine —20 grains of the former and one-half grain of the latter; but unfortunately, cases of acute spinal inflammation are not often seen at their beginning. The attempts to jugulate an inflammation can therefore be very rarely made with success. The cases of chronic inflammation are relatively more frequent. They succeed to the acute; they may arise *de novo*. Hyperplasia of the neuroglia, granular degeneration of nerve fibers and cells, fatty and granular degeneration of the intima of the vessels, and crowding of the perivascular lymph spaces with leucocytes, are the most important initial changes, and these lead to various inflammatory and atrophic lesions. The various scleroses belong to the group of chronic inflammatory affections—such as antero-lateral and posterior spinal sclerosis. The alterations extend over many years, but they are, properly speaking, of the chronic inflammatory type. It must be obvious to you that some of the most important therapeutical questions are concerned in the management of these affections. The means employed are partly topical; partly systemic. A daily morning and evening hot douche to the spine, of fifteen minutes' duration. I have found it to be exceedingly effective.

In the absence of suitable appliances for the douche, a sponge dipped in hot water and passed over the spine rapidly for fifteen minutes at a time may be accepted as its equivalent. You may regard this as trivial, but I assure you the day of small things has not yet passed. The importance of physical impressions on the peripheral nerves is indeed very great. Strumpf, the well-known neurologist of Düsseldorf, has lately got remarkable curative results in posterior spinal sclerosis by cutaneous faradization, by excitation with the faradic brush of the skin of the back, especially of the spine and of the limbs. This method consists in passing the faradic brush for a half hour at a time thoroughly over the parts in which the lesion is known to exist, and over those parts in which symptoms are felt. You will probably feel inclined to ask, How can cutaneous faradization effect the cure of a disease which has hitherto resisted the most efficient treatment? It may be quite impossible to give an adequate explanation, but it is certain that peripheral irritation, if restrained within proper limits, has a remarkable curative effect on the condition of internal organs so situated as to be in anatomical relation to the site of irritation. Thus, Strumpf has found that cutaneous faradization of one side will cause an increase of the temperature in the corresponding position on the other side. Erb strongly advocates the use of the rubbing wet-pack in chronic myelitis. He does not advise cold or hot water, but merely tepid. The patient, enveloped in a sheet wrung out in tepid water, is gently rubbed with the sheet *in situ*. To the same class of actions belongs massage, but this alone has, in my experience, been disappointing. If the application can be confined to the gentlest titillation of the cutaneous nerves, good may come of it; but in the spinal trouble I am referring to, all violent rubbing and kneading apparently does mischief. Granville's *percutier*, lightly used, has a good effect also. It is a matter within the range of everybody's experience that the most painful inflamed surface will be anæsthetized by the gentlest friction if persistently employed. It is probable, therefore, that the good effects of peripheral applications in spinal affections are largely due to the communication of very gentle impulses originated at the periphery, in the sensory terminals.

Besides these topical applications which act through the sensory nerves, there is a local remedy which acts directly on the cord—galvanism. Since it has been shown that the galvanic current penetrates through the bony envelope of the cord, the only question is as to the mode of applying the electrodes. Strangely enough, opinions are yet divided as to the effect of direct or inverse currents. My own conviction is, that the view of of Onimus and Legros is substantially correct. According to them, the descending galvanic current increases the activity of the circulation in the part acted on, by stimulating the vermicular contractions of the organic muscular fiber of the vessels. The result of such increased vermicular motion, must necessarily be a more active circulation, and more rapid interchanges between the blood and the tissues. A descending galvanic current must therefore promote the nutrition of parts, if we admit the correctness of the views enunciated by Onimus and Legros. It is therefore, in chronic spinal affections that we have a right to expect the best results from galvanic treatment. As the resistance offered by the bones of the spinal column is so great, the electro-motive force of the galvanic battery must be sufficient to overcome it.

To treat such cases efficiently, not only must the number of elements be large, but the resistance within the battery must be nearly that of the part of the body acted upon, to avoid the shock and burning pain. The electrodes should be of large, well-moistened sponges—one placed on the nape of the neck, the other on the sacrum. As the strength of current required for these spinal applications is so great, unless the sponges are large and well-moistened, very severe burning will be produced. The number of elements required will be from thirty to sixty, or the strength in *milleampères*, from ten to twenty. There should be a daily *séance* of ten to fifteen minutes' duration, and the treatment should be continued over many months to obtain permanent results. It is the neglect of these measures, inattention to details, and impatience over delays that have led to so much disappointment in the application of galvanism. I am, from personal observation, fully able to confirm the high estimate placed by Erb on the method of treating chronic spinal affections. Neither galvanism nor any other remedy can restore lost parts; hence, when the anatomical elements are destroyed, function must ever after be imperfectly performed.

I must now call your attention to the most useful internal remedies in chronic cases of spinal inflammation. The best results have been obtained from the so-called metallic tonics, notably nitrate of silver, but the danger of staining and of causing gastric ulceration are serious objections to the persistent administration of silver salts. In the scleroses, and in connective tissue hyperplasia of organs in general, I have seen excellent results from the internal use of the chloride of gold and sodium. This may be given in a granule containing one-twentieth of a grain three times a day. The corrosive chloride of mercury has similar effects, but it does not seem to me to be equal in curative power to the gold chloride.

There are so many ways, owing to the almost universal use in domestic life of the noxious metals, for slow metallic poisoning to occur, that the affections thus produced are probably much more numerous than we are now aware of. A typical example of locomotor ataxia came into my hands, in the person of a water gilder. Syphilis is now held to be the chief pathogenetic factor in this disease, but there are two modes in which this relation exists—direct and indirect. Syphiloma occurs when the first cutaneous lesions appear, but especially during the time of the older constitutional symptoms. The first are comparatively mild, the second consist of gummata, etc. These lesions are direct—that is, they belong to the ordinary course of development—of evolution of the disease. The indirect arise because of the cachexia—the lowered vital resistance of the organism, produced by the long-continued operation of the syphilitic disease and of the remedies used to remove it. In the former, or the direct syphilitic lesions, the specific remedies are curative; in the latter or indirect, they rarely do good; in fact, their administration is adding insult to injury, in many instances.

I have long entertained the notion that the utility—the remarkable utility—of iodide of potassium in some cases is due to one of two conditions: to an overlooked syphilitic infection; to metallic poisoning. This is so certainly a fact, that in doubtful cases I advise the use of full doses of iodide of potassium as a preliminary to further treatment. In all distinctly specific cases there can be no possible doubt in regard to the efficacy of anti-syphilitic treatment. Now let me tell you a fact not generally known, or, if known, not sufficiently appreciated: Mercury, in syphiloma of the nervous system, tertiary in type, is, in some instances, quickly curative when the iodides fail utterly. *When there is reason to suspect mineral poisoning, and in all doubtful cases, the iodides should be administered in full doses, either tentatively or as the major treatment.*

The third group, from the therapeutical point of view, consists of these spinal disorders not due to a recognizable inflammatory process, but to some change in the function of nutrition. In this position may be placed the changes that are senile, whether of time or prematurely. The most important therapeutical point in these cases is to supply the material in which the tissues are deficient. A combination of the lime salts—the phosphate especially—with a fat, cod-liver oil, is most useful in these cases, but good results can be reached only by persistent use of the means of treatment. In these cases of senile degeneration, much good is accomplished by the use of the salts of ammonia to prevent the formation of thromboses, or to effect their solution if formed. Strychnia and quinine, to stimulate the organic functions, render an important service also.

The subcutaneous use of strychnia is often remarkably effective in all of the chronic spinal affections characterized by loss of muscular power. It is, of course, inadmissible in those stages of these maladies having an active state of the local circulation—in all acute cases, in chronic cases with acute exacerbations. The quantity to use in this way ranges from 1-60 to 1-30 grain daily, once, or on alternate days. The hypodermatic injection of strychnia may, indeed, serve as a means of distinguishing the character of the spinal trouble. It increases the paralytic symptoms when an inflammatory condition is present, and improves the functional and chronic diseases.

Galvanism, applied as already pointed out, to stimulate the spinal circulation, and faradism at the periphery, contribute to the nutrition of the cord by promoting the activity of the circulation in general. By a proper combination of these expedients, we can often effect very decided improvement.

As you will observe, I have not alluded to the treatment of the large group of so-called functional disorders of the spinal cord. The consideration of this is a sufficiently fruitful topic of itself for a lecture—for many lectures, indeed—and hence I must postpone it to a more convenient season.—*Weekly Medical News.*

